# Real-Time 3D Multi-Object Detection and Localization Based on Deep Learning for Road and Railway Smart Mobility

**DOI:** 10.3390/jimaging7080145

**Published:** 2021-08-12

**Authors:** Antoine Mauri, Redouane Khemmar, Benoit Decoux, Madjid Haddad, Rémi Boutteau

**Affiliations:** 1 Normandie Univ, UNIROUEN, ESIGELEC, IRSEEM, 76000 Rouen, France; benoit.decoux@esigelec.fr; 2 Haddad is with SEGULA Technologies, 19 rue d’Arras, 92000 Nanterre, France; madjid.haddad@segula.fr; 3 Normandie Univ, UNIROUEN, UNILEHAVRE, INSA Rouen, LITIS, 76000 Rouen, France

**Keywords:** object detection, localization, distance estimation, object dimensions, object orientation, 3D bounding box estimation, 3D multi-object detection, multi-modal dataset, deep learning, smart mobility

## Abstract

For smart mobility, autonomous vehicles, and advanced driver-assistance systems (ADASs), perception of the environment is an important task in scene analysis and understanding. Better perception of the environment allows for enhanced decision making, which, in turn, enables very high-precision actions. To this end, we introduce in this work a new real-time deep learning approach for 3D multi-object detection for smart mobility not only on roads, but also on railways. To obtain the 3D bounding boxes of the objects, we modified a proven real-time 2D detector, YOLOv3, to predict 3D object localization, object dimensions, and object orientation. Our method has been evaluated on KITTI’s road dataset as well as on our own hybrid virtual road/rail dataset acquired from the video game Grand Theft Auto (GTA) V. The evaluation of our method on these two datasets shows good accuracy, but more importantly that it can be used in real-time conditions, in road and rail traffic environments. Through our experimental results, we also show the importance of the accuracy of prediction of the regions of interest (RoIs) used in the estimation of 3D bounding box parameters.

## 1. Introduction

To improve safety in intelligent mobility and to make driving more autonomous, vehicles must have a better perception of their environment. This perception must guarantee good detection of objects, but more generally good interpretation of the scenes. In rail mobility, the problem is quite similar to that in the road sector. In these two types of environment, the objects of interest are vehicles, pedestrians, buses, cyclists, trees, etc. However, while the road sector is widely covered in the scientific literature, this is not the case for the rail sector. This is in part due to the lack of specific datasets. Reliable perception must guarantee completion of at least three essential tasks: object detection, localization, and tracking. An accurate and reliable estimation of the depth could significantly increase safety, by allowing the estimation of the distance between the vehicle and the detected objects, thus allowing the prevention of collisions. Other information about objects such as their dimensions and orientation is also very useful for safety. The estimation of those parameters is consequently an important task that should be performed by an advanced driver-assistance systems (ADAS).

Measurement of the distance to objects is generally based on a range of complementary sensors: radar [1], LiDAR [2], or time-of-flight (ToF) camera [3]. However, these methods have some limitations because they are often expensive and bulky. We focus in this paper rather on the use of vision-based techniques for object detection in 3D, which combine the detection of objects and their localization in 3D. In recent years, methods based on convolutional neural networks (CNNs) have been explored for detecting and locating objects in 3D space with only images as inputs. One of the main advantages of these methods is the reduction of sensor costs. Light detection and ranging (LiDAR) and radar are replaced by a standard camera, which is easy to integrate and is inexpensive. Many approaches have been proposed in the literature. However, we note a deficit in the evaluation of these approaches in realistic environmental conditions, specifically in rail traffic environments.

The main contributions put forward in this paper are the following:

- A new method for a real-time multi-class 3D object detection network that is trainable end-to-end. We leverage the popular real-time object detector You Only Look Once (YOLO) v3 for regions of interest (RoIs) prediction used in our network. Our methods allow the following predictions:
  1. Object 2D detection;
  2. Object distance from the camera;
  3. Object 3D centers projected on the image plane;
  4. Object 3D dimension and orientation.
  With these predictions, our method is able to draw 3D bounding boxes for the objects.
- A new photo-realistic virtual multi-modal dataset for 3D detection in the road and railway environment using the video game Grand Theft Auto (GTA) V. The GTAV dataset includes images taken from the point of view of both cars and trains.

Our approach is mainly based on the contribution of an object detection method taking into account two significant criteria: accuracy and real-time for both road and rail environments. While other methods in the state-of-the-art focus mainly on the accuracy of 3D object detection, in railway applications we note that no work is published on 3D object detection. No datasets are currently available in the state-of-the-art that include railway scene data with the ground truth. We use an approach based on YOLOv3 for real-time 3D object detection and we use the GTAV game for the creation of the virtual railway dataset (instead of using a simulator, for more graphical fidelity) for training and validation of our method. The remainder of this paper is organized as follows. Section 1 introduces the paper. In Section 2, we review the 3D-object-detection-related work in which we will present methods based on depth sensors as well as methods based on monocular images. In Section 3, we describe in more detail our approach for real-time 3D multi-object detection and localization. Experimental results are presented in Section 4. Finally, the conclusions and future directions are outlined in Section 5.

## 2. Related Work

Many works have been carried out on 3D object detection. Among these works, we can find methods based on high-precision depth sensors such as LiDAR and methods based only on images from a single camera.

### 2.1. 3D Object Detection from a Depth Sensor

Among the methods based solely on depth sensor inputs, we can distinguish the methods using only a point cloud from LiDAR sensors like PointNet [4] and Pipeline based on graph convolutional networks (PointRGCN) [5]. In PointNet, a deep network architecture is presented. This network allows performing tasks such as object classification, part segmentation, and semantic segmentation from a 3D point cloud from a depth sensor. The PointNet network is divided into two sub-networks: the first one is the classification network and the second one is the segmentation network. The classification network takes the points from the point cloud and performs feature transformation to extract global features. These global features are then passed through a multi-layer perceptron (MLP) to obtain the classes to score from the classification. The global features are then also fed to the segmentation network to obtain the semantic segmentation. This method has been trained on the indoor dataset presented in [6] but has not been trained on outdoor datasets for road applications.

However, PointRGCN has been trained for road applications on the KITTI road dataset [7]. It introduces a graph-based 3D object detector based on graph convolutional networks (GCNs). The method relies exclusively on 3D point clouds from a LiDAR sensor to perform the 3D object detection task. This method is based on three modules: a region proposal network (RPN) module, a graph-based network for feature extraction, and a graph-based network for context aggregation.

We can also denote methods that use as the input the point cloud from a LiDAR and RGB camera to improve 3D detection. In [8], the proposed method puts forward a new architecture for the RPN used for predicting the RoIs. The method uses feature extractors to create the feature maps for both RGB input images and LiDAR point clouds. The feature maps are then fed to the multimodal fusion RPN introduced in this paper, which leverages the data from both inputs to perform the 3D bounding box proposals. These proposals have their dimension refined in the detection network and their orientation and class predicted. This network has also been trained in road environments through the KITTI dataset.

LiDAR-based methods have the advantage of being the most accurate methods available for 3D object detection. However, they require a dense point cloud from an expensive depth sensor such as a LiDAR. This limits the development of datasets that are necessary for training, validation, and inference.

### 2.2. 3D Object Detection from Monocular Images

In recent years, we have seen the emergence of CNN-based methods for 3D detection. These methods enable 3D detection from simple RGB images. In [9], the bounding boxes are predicted by using prior 3D boxes with typical object sizes on the ground plane. These boxes are then projected onto the image plane and the scores are computed by using features like the class semantic, instance semantic, contour, object shape, context, and location prior. The final boxes are obtained after the application of non-maximum suppression (NMS)-based methods.

2D-detector-based methods [10,11,12] use separately trained 2D detectors for feature extraction and RoI prediction, which are used to obtain aligned features that are then fed to a 3D parameter regressor. Ref. [10] proposes a 3D parameter regression network from RoI-aligned features with a new hybrid discrete-continuous loss for orientation prediction. This allows leveraging the geometric constraints given by the 2D bounding boxes to increase the accuracy of the 3D bounding boxes. This method turns the regression of the object angle into a hybrid task with a classification part as well as a regression part. It is shown to increase the precision of the angle prediction. For the classification task, the angles are separated into bins, and then the network predicts the confidence score for an observed angle to belong to a specific bin. The network also computes the cosine and the sine of the offset angle from the bin centers (regression). Another part of the model regresses the dimension of the object. This new loss function provides a better prediction of the object’s angle compared to the simple regression. This method still requires an RPN-based object detector for extracting the features as well as obtaining the RoIs used for feature alignment.

In [11], a CNN-based approach for 3D object detection and tracking is proposed. This method uses a Region Based Convolutional Neural Network called FasterRCNN to first predict the 2D bounding boxes along with the object class, and then predict its projected 3D center on the image plane. For each 2D box, the dimensions, orientations, and distance along the Z-axis from the camera are predicted to obtain the oriented 3D bounding boxes. The tracking across an image sequence is performed by two long short-term memory (LSTM) layers. These temporal layers produce a robust linking across the frames and allow for further refinement of the 3D bounding boxes. The whole method is trained and evaluated on a custom dataset, but the authors also provide 3D detection quantitative results on the KITTI dataset.

In [12], a deep coarse-to-fine many-task (deep MANTA) CNN is introduced for simultaneous vehicle detection, part localization, visibility characterization, and 3D dimension estimation, from monocular images. It is based on a two-step process: the first step outputs scored bounding boxes associated with vehicle information, and the second uses these outputs and a 3D vehicle dataset to recover 3D orientations and locations.

These methods provide relatively accurate results but still fall short of methods that take point clouds from depth sensors as the input. These methods also do not focus on the real-time aspect that is essential for an embedded system application for both road and rail safety. Finally, we can denote the complete absence of methods evaluated in railway environments due to the lack of adequate datasets. Our work aims at filling this gap in the state-of-the-art by proposing a new real-time 3D detection method trained and evaluated on our own virtual road/rail GTAV-based dataset, as well as on the KITTI dataset.

## 3. Our Realtime 3D Multi-Object Detection and Localization Network

**Figure 1:**
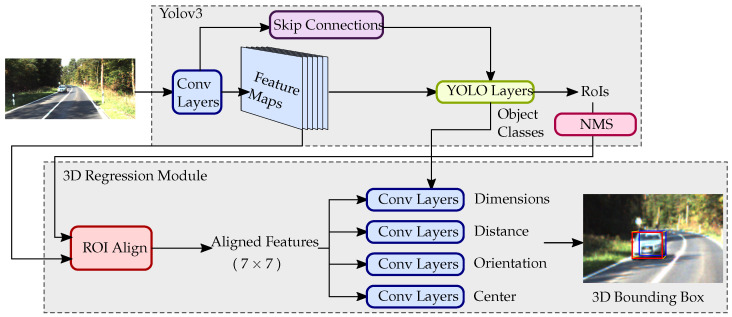
Illustration of our 3D bounding box detector. A single RGB image is used as the input for our method; the shared convolutional features are then extracted by the network backbone, Darknet-53. We leverage the proven 2D object detector, YOLOv3, to perform the RoI and object class prediction. We then extract the RoI features by using the feature alignment used in [14]. The 3D bounding box parameters are predicted by our CNN’s parameter prediction, and finally the 3D bounding box is drawn on the image.

The aim of our method is to retrieve 3D bounding boxes from monocular RGB images while keeping the computing time low to be compatible with real-time constraints. Like most of the works related to 3D object detection from a single image presented in the previous section, we rely on RoIs provided by a 2D object detector to estimate the oriented 3D bounding boxes of the objects. Unlike the other methods for 3D detection from a monocular image, which rely on a separate RPN-based network like faster RCNN to perform the 2D detection, our method is based on the single-stage detector YOLOv3 [13] to perform the 2D bounding box predictions, and our network shares the same feature extractor between the 2D detector and the 3D detector. Our network architecture allows the memory consumption to be greatly reduced, while outperforming other state-of-the-art methods in terms of computing time, at the cost of slightly lower precision. Furthermore, our method is trained end-to-end, while other models require the 2D detector to be trained separately, thus reducing the training time and cost of our method. Our detection pipeline is described in Figure 1.

### 3.1. 3D Bounding Estimation

The 3D bounding box of an object can be described by its center position relative to the camera $T={\left[x\;y\;z\right]}^{⊤}$ its dimensions D=[w,h,l] and its orientation R(ϕ,θ,ψ) characterized by the elevation, the azimuth, and the roll angles. Given K the matrix of intrinsic parameters of the camera and $X_{o}={\left[x_{o}\;y_{o}\;z_{o}\;1\right]}^{⊤}$ a 3D point in the object coordinate system, the projection of this point onto the image plane $x_{\mathrm{im}}={\left[u\;v\;1\right]}^{⊤}$ is given by:

(1) $$x_{\mathrm{im}}=K\cdot \left[\begin{array}{cc} R & T \end{array}\right]\cdot X_{o}.$$

By considering the origin of the object coordinates to be the center of the 3D bounding box, the coordinates of the 3D bounding box are $X_{1}=\left[\begin{array}{ccc} w/2 & h/2 & l/2 \end{array}\right]$, $X_{2}=\left[\begin{array}{ccc} w/2 & -h/2 & l/2 \end{array}\right]$, …, $X_{8}=\left[\begin{array}{ccc} -w/2 & -h/2 & -l/2 \end{array}\right]$. The 3D bounding box coordinates in the image can then be obtained using Equation (1).

### 3.2. Parameters to Regress

**2D object detection.** In our work we use YOLOv3 to perform the 2D object detection. YOLOv3 performs the object class classification cls as well as the bounding box parameters *b* (position and dimension). The bounding box predictions are then used as RoIs for the Feature Align function to extract the features for each RoI, which are then fed to the rest of the network for predicting the 3D bounding box parameters.

**Object center prediction.** In this work, we assume the center of the 3D bounding box to be the 3D center of the object. Predicting the center of the 3D bounding boxes of the objects is therefore equivalent to predicting their position $X_{o}={\left[x_{o}\;y_{o}\;z_{o}\;1\right]}^{⊤}$. In order to increase the accuracy of this prediction, we aim at predicting the projected 3D center on the image plane. Instead of predicting directly the coordinates of the object center on the image plane, we use cues from the 2D bounding box prediction and we predict the offset position in pixels of the object center from the 2D bounding box center $\tilde{c}=\left[\begin{array}{cc} \tilde{cx} & \tilde{cy} \end{array}\right]$. We are therefore reducing the variance of the prediction, making it easier for the algorithm to learn. The object center $X_{o}$ is computed by using the object distance estimation on the Z-axis and the inverted calibration matrix $K^{-1}$.

**Object distance estimation.** In order to determine the center of the 3D bounding box, it is necessary to determine its position on the Z-axis of the camera coordinates. For each RoI from the object detector, we predict the object’s center distance $\tilde{z}$ in meters.

**Object dimensions.** Instead of directly predicting the object’s dimensions in meters, we use the fact that the dimensions of objects have a very low variance among the same class (car, truck, etc.). Therefore, we choose to use the mean dimensions for each object class as a strong prior for the prediction of the dimensions.

**Figure 2:**
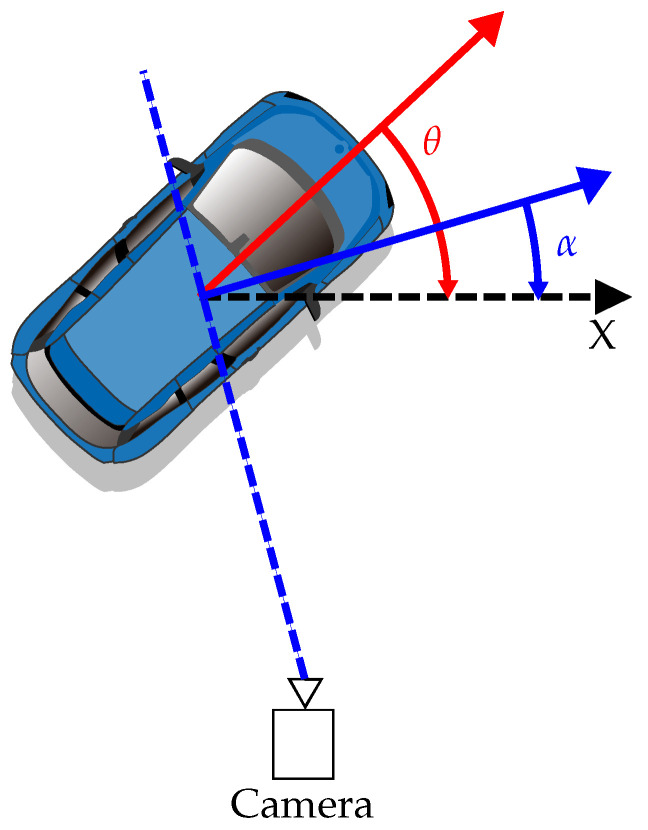
Illustration of the object azimuth θ and its observed orientation α. The local orientation is retrieved by computing the angle between the normal to the ray between the camera and the object center and the X-axis of the camera. Given that we are using left-hand coordinates, the rotation is clockwise. Our method estimates the observed orientation and θ can be obtained using Equation (2).

**Object orientation.** In our work, we assume that only the azimuth (noted θ) matters for application on the road environment, and thus we do not predict the orientation characterized by the elevation and roll and we set ϕ=0 and ψ=0. Since the same azimuth can lead to multiple observed orientations from the camera point of view (see Figure 2), we cannot predict directly the angle θ. Instead, we predict the observed angle α and retrieve the global orientation θ using Equation (2):

(2) $$\theta =\alpha +arctan(\frac{x}{z}).$$

Following the work of [10], instead of considering the angle prediction as a regression problem, we adopt a hybrid classification/regression approach. We divide the possible angles into 2 bins; we then perform a classification task to predict in which bin the object angle is located. Then, we regress the difference between the bin center and α.

### 3.3. Losses

In this subsection, we present the loss calculation of our method. The loss is specified in Equation (3):

(3) $$L=L_{yolo}+k_{1}\times L_{center}+k_{2}\times L_{distance}+k_{3}\times L_{dim}+k_{4}\times L_{orient}.$$

In our loss calculations, we use the same loss as YOLOv3 for 2D detection and class prediction. We also use $k_{i\in [1,\ldots ,4]}$ as weights for the losses. With $c_{k}=\left[\begin{array}{cc} cx^{k} & cy^{k} \end{array}\right]$ the ground-truth center of the object *k* in pixels, ${\mathrm{Rc}}_{k}=\left[\begin{array}{cc} Rcx_{k} & Rcy_{k} \end{array}\right]$ the center of the RoI for the object *k*, *N* the number of objects, and ${\tilde{c}}_{k}$ the prediction from our network, the center loss is written in Equation (4):

(4) $$L_{center}=Mean\left(\frac{1}{N}\sum_{k=1}^{N}\left|c_{k}-{\mathrm{Rc}}_{k}-{\tilde{c}}_{k}\right|\right).$$

Assuming $z_{k}$ to be the ground-truth distance of an object k, *N* the total number of objects, and ${\tilde{z}}_{k}$ the distance prediction from our method, the distance loss is then calculated using the L1 loss and is detailed in Equation (5):

(5) $$L_{distance}=\frac{1}{N}\sum_{k=1}^{N}\left|z_{k}-{\tilde{z}}_{k}\right|.$$

With $d_{k}=\left[\begin{array}{ccc} dx & dy & dz \end{array}\right]$ the ground-truth dimensions of an object *k* in meters, $dd_{k}$ the mean dimension for the class of object *k*, *N* the number of objects, and ${\tilde{d}}_{k}$ the prediction from our method, the distance loss is detailed in Equation (6):

(6) $$L_{dim}=Mean\left(\frac{1}{N}\sum_{k=1}^{N}\left|d_{k}-{\tilde{d}}_{k}-{\mathrm{dd}}_{k}\right|\right).$$

Finally, assuming $\alpha_{k}$ to be the ground-truth observed angle of object *k*, *N* the total number of objects, and ${\tilde{\alpha }}_{k}$ the prediction, we use the smooth L1 loss as the orientation loss and the loss is written in Equation (7) with β=1:

(7) $$L_{orient}=\frac{1}{N}\sum_{k=1}^{N}e_{k},$$

where

$$e_{k}=\left\{\begin{array}{cc} 0.5{\left(\alpha_{k}-{\tilde{\alpha }}_{k}\right)}^{2}/\beta , & \mathrm{if}\;|\alpha_{k}-{\tilde{\alpha }}_{k}|<\beta \\ |\alpha_{k}-{\tilde{\alpha }}_{k}|-0.5\times \beta , & \mathrm{otherwise}\; \end{array}\right..$$

## 4. Experimental Results

### 4.1. Training Details

**KITTI.** We trained our method on the KITTI dataset dedicated to 3D detection on the same training split used by the authors of [10] containing half of the samples and we performed the evaluation on the other samples. This dataset offers over 7000 training image annotations for 2D bounding boxes, object position (XYZ), object dimensions, object azimuth, and observed orientation for 3 different object classes (car, bicycle, person). Optimal results (optimal-fitting) for our method were achieved after 130 epochs.

**GTA.** Inspired by previous work on database creation using images from the video game Grand Theft Auto V [15], we created our own hybrid database of road and rail images. This new dataset allows us to overcome the problem of having a railway database with a ground truth rich enough to allow 3D bounding box learning for cars, trucks, pedestrians, and motorcycles. The training of our method was conducted on a split containing road and railway images. The evaluation was then conducted on the validation split of the database containing only railway images. The dataset contains a total of more than 10,000 images. The training on this dataset was performed on 50 epochs.

**Training.** For estimating the 3D bounding box, we used a pre-trained YOLOv3 model trained on the COCO dataset to reduce the training time. The training on both datasets (KITTI and GTAV) was performed with an image resolution of 512×512 pixels with a batch size of 64. We used the one-cycle learning rate scheduler as proposed in [16] for controlling the momentum and the learning rate during training. The optimal maximum learning rate was determined using the method described in the same paper [16]. For our method, we used a peak learning rate of $7\times 10^{-4}$ with a weight decay of $1\times 10^{-3}$. Through trial and error we also determined the loss weights and we set $k_{1}=1$, $k_{2}=5.1$, $k_{3}=70$, $k_{4}=110$. We also chose the Adam optimizer with a weight decay of $7\times 10^{-5}$ for training optimization.

### 4.2. Evaluation

**2D detection.** For evaluating the performance of the 2D detection, we used the metrics described by the authors of YOLOv3 on each class of the dataset. Given that the regression of the 3D bounding box parameters relies on accurate RoIs and classes for the objects, evaluating the precision of the 2D detector is a necessity. The error metrics are the average precision (AP), the recall (R), the mean average precision (mAP), and the F1 score.

**Distance estimation.** For our distance estimator evaluation, we used the same evaluation as was used for image-level depth estimation methods. The metrics used include absolute relative error (Abs Rel), the squared relative error (SRE), the root-mean-square error (RMSE), the logarithmic RMSE (log RMSE), and the percentage of bad matching pixels (BMP). Let $z_{gt}$ and $z_{pd}$ be, respectively, the ground truth and predicted distance of the object *i*, calculated using Equation (8), where $\delta =1.25^{k}$:

(8) $$\alpha_{k=\left[1\ldots 3\right]}=max\left(\frac{z_{gt}}{z_{pd}},\frac{z_{pd}}{z_{gt}}\right)<\delta^{k}.$$

**Dimensions.** The dimension prediction evaluation is performed using the dimension score (DS) described by the authors of [11]. With $V_{pd}$ and $V_{gt}$ the predicted and ground truth volume of the object, the DS is computed using Equation (9):

(9) $$DS=min(\frac{V_{pd}}{V_{gt}},\frac{V_{gt}}{V_{pd}}).$$

**Object center.** The object center predictions are evaluated with the center score (CS) as described in [11]. Assuming *x* and *y* are the projected center coordinates in pixels and *w* and *h* the width and the height of the 2D bounding box, CS is computed with Equation (10):

(10) $$CS=(2+cos\left(\frac{x_{gt}-x_{pd}}{w_{pd}}\right)+cos\left(\frac{y_{gt}-y_{pd}}{h_{pd}}\right))/4.$$

**Orientation.** For evaluating the orientation predictions, we use the orientation score (OS) as described in the KITTI benchmark. OS is calculated using Equation (11):

(11) $$OS=(1+cos\left(\alpha_{gt}-\alpha_{pd}\right))/2.$$

### 4.3. Results

**Figure 3:**
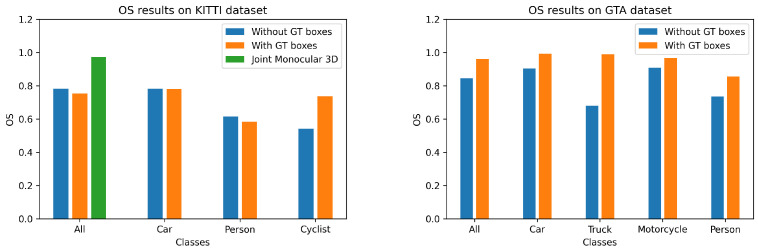
In these graphs, we compare the orientation score obtained by our method (with or without ground truth box) on the different dataset classes; we also include the results of the “3D joint monocular” method (which also uses ground truth boxes). We can see that our method has a lower orientation score when we do not use Ground truth (GT) bounding boxes. This can be explained by the fact that the boxes used for feature alignment during inference are the same as those used during training.

**Figure 4:**
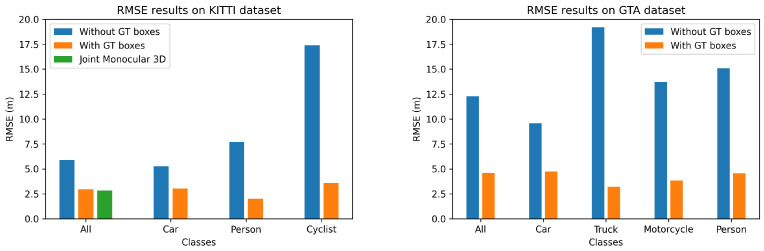
In these graphs, we compare the depth RMSE obtained by our method (with or without ground truth box) on the different dataset classes; we also include the results of the “3D joint monocular” method (which also uses ground truth boxes). We can see that our method obtains a higher RMSE error when we do not use GT bounding boxes. This can be explained by the fact that the boxes used for feature alignment during inference are the same as those used during training. We can also see that there is a significant loss in accuracy on smaller classes such as bicycles or people when our method predicts RoIs using YOLOv3 instead of using ground truth. This can be explained by the fact that variations in the prediction of RoIs have a greater impact than for larger classes like cars.

**Figure 5:**
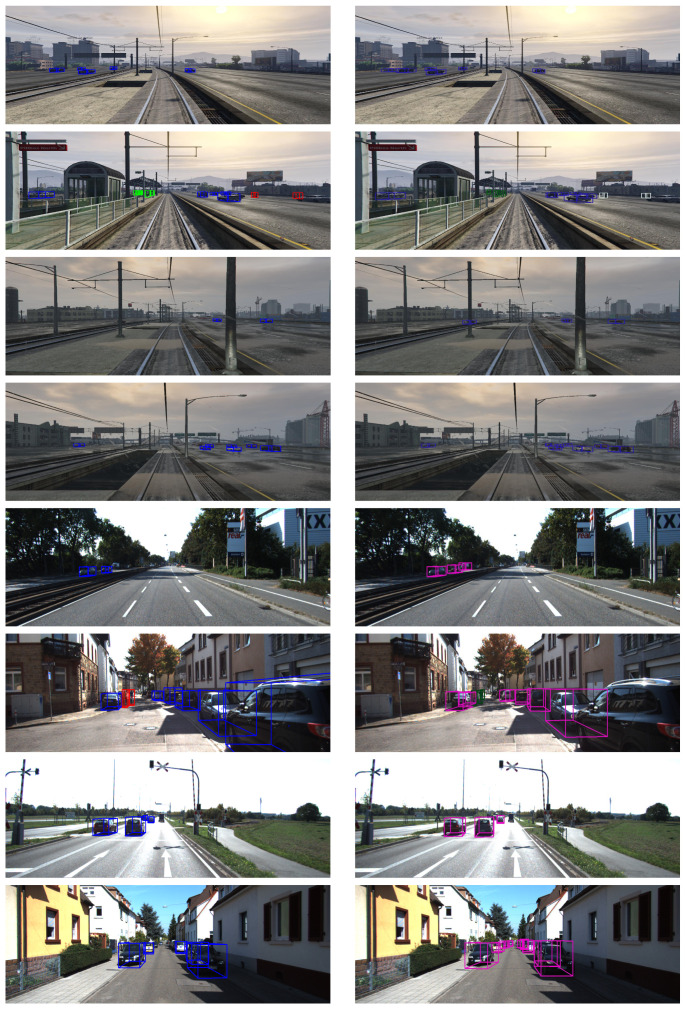
Qualitative results of our method were obtained through KITTI and our GTAV datasets. These images were extracted from the validation split of each dataset. The RoIs used for predicting the 3D bounding box parameters were computed through YOLOv3. 4 top lines: results obtained for GTAV dataset (left column: ground truth; right column: prediction), 4 bottom lines: results obtained for KITTI dataset (left column: ground truth; right column: prediction).

The quantitative results of our method on both KITTI and GTAV datasets are presented in Table 1 and Figure 3 and Figure 4. We can see that although our method offers results that are slightly lower than the state-of-the-art methods, the lightweight architecture of our network allows us to perform real-time 3D detection, which is not possible with the other state-of-the-art methods. We have also added the qualitative results of our method under both GTAV and KITTI datasets in Figure 5.

In order to carry out a 3D object detection, our method requires 2 stages: 1. The first level allows extraction of the image features to predict the RoIs of the objects and their classes. 2. The second level of our CNN network uses both extracted features and the RoIs to align the features and to predict the 3D parameters of the objects. As for the method presented in [10], this presents an approach to improve the prediction of orientation during 3D detection while our approach predicts not only the objects’ orientation, but also their distance, their 3D centers, and their dimension for a full 3D object detection. The architecture of the network presented in [10] has only one stage (the second stage in our approach) for 3D prediction and requires adding the first stage using a 2D object detector (faster RCNN, YOLO, Single-Shot Detector (SSD), etc.). Moreover, the results presented in [10] focus on the evaluation of the orientation under the KITTI dataset and do not include an evaluation dedicated to 3D object detection. Our approach predicts not only 2D object detection, but also the distance of the objects from the camera, their 3D center, their orientation, and their dimension. All these predictions are integrated into an “all-in-one” network to predict 3D objects. For this reason, we have presented in Table 1 quantitative results of our method on both KITTI and GTA datasets compared to [11], and in Table 2 some experimental results for different object classes on both KITTI and GTAV compared with [11].

We conducted our experiments on both datasets using our method with either the RoIs coming from YOLOv3 or coming directly from the ground truth. The results show that the accuracy of our method, especially for distance estimation, is dependent on the accuracy of the RoI prediction. Thus, we can see that our method, when the ground truth RoIs are used, has a precision close to the state-of-the-art methods. However, when we use the RoIs predicted by YOLOv3, the accuracy is significantly lower, which can be explained by the fact that RoIs for the same target can vary in size and shape, which makes learning the distance more difficult. This problem is mitigated when the method is evaluated using the same fixed RoIs as those used for learning, which explains the good performance of the state-of-the-art methods and of our method with the ground truth RoIs. In our 3D object detection approach, we use images and not videos, so we do not have problems related to full RoI selection where objects change position in video sequences. We also note through our results that the OS is relatively low; this can be explained by the fact that our method struggles to distinguish the front from the back of the detected objects. However, when plotting the 3D bounding boxes on the image, this problem is mitigated. Our 3D object detection method, although being one of the fastest, does not yet reach the level of accuracy of state-of-the-art methods. This is because the variation of RoIs during the 2D prediction phase leads to a loss of accuracy when predicting the 3D parameters. We are currently working on a new method that will use 2D/3D anchor boxes to replace the prediction part of the RoIs and thus avoid the loss of accuracy when these vary. Predicting the 3D parameters of an object from a 2D image is a complex problem. By predicting the 3D center projected on the 2D image, we can improve the prediction of the 3D center. Our network can thus use the information related to the appearance of the object to deduce the position of the object’s 3D center on the image. By combining this information with the prediction of the distance of the object from the camera and the object calibration matrix, we can obtain a prediction of the object’s 3D center. With this approach, we can increase the accuracy of the object 3D prediction; however, it is still not as accurate as using LiDAR data. The accuracy is also reduced when the object is partially hidden by any other obstacle present in the scene. Additional evaluations of our methods were conducted for the different classes of objects present on the two datasets (see Table 2). These results show that on both KITTI and GTAV datasets, the object class with the highest precision is the Car class. This is explained by the fact that the Car class is the majority class on both datasets and by the fact that a car represents a relatively large target (unlike a person or a cyclist) making detection easier.

We have used YOLOv3 as the first stage because at the time of our network design, YOLOv3 was the most responsive/accurate real-time 2D object detector (better than Faster RCNN, SSD, etc.). YOLOv4, which was published in 2020, has made improvements on the backbone architecture of YOLOv3 (moving from Darknet53 to Darknet53CSP) due to improvements in data augmentation during the training process. During the development of our network, we have tested the different improvements related to the data augmentation on our network and, although they improve the precision for 2D object detection, they deteriorate and decrease the quality of 3D detection. Therefore, we chose not to make these improvements on our network and kept YOLOv3 as a main approach in the first stage.

Finally, we conducted experiments on the computation time and memory consumption of the different models, which can be found in Table 1. Since the method proposed by [11] is separated into three modules (RoI prediction, 3D parameter regression, and tracking), we obtained the computation time by adding one of the forward passes of the RoI prediction and one of the 3D parameter predictions. The memory consumption was obtained similarly. The results of this experiment show that our method’s single network architecture allows us to greatly reduce the computation time as well as reducing the memory footprint suitable for embedded system real-time applications. The experiment was conducted on an Nvidia RTX 3080. Our approach is innovative for at least two significant reasons. Firstly, our new approach of 3D object detection is adapted in real-time to real navigation and traffic conditions. We tested it with our test vehicle in Rouen and Le Havre (two large cities in France). The real-time 3D object detection improved the quality of environment perception, which improved the quality of both decisions and actions (obstacle avoidance, pedestrian detection, maintaining a safe distance, etc.). Our approach allows 2D detection, object center prediction, object distance prediction from the camera, object 3D center prediction, 3D object dimension, and 3D object orientation. This means that our method is based on a new network that is trainable end-to-end, which facilitates the training process. Our approach is one of the fastest and lightest methods compared to the state-of-the-art methods. This makes it the most suitable 3D object detection method for embedded applications such as autonomous vehicles (cars and trains). Indeed, the other approaches in the state-of-the-art do not put the real-time aspect as a high priority. Secondly, we have developed a new GTAV virtual multi-modal dataset (both camera and LiDAR) with ground truth for both road and rail environments. The GTAV dataset includes images taken from the point of view of both cars and trains. This is another innovation of our approach because no dataset exists today for smart rail mobility, whether a real or a virtual dataset. However, we are developing a second rail dataset that is a real dataset. This one has been collated in two French cities (Rouen and Le Havre).

## 5. Conclusions

In this paper, we have introduced a new method of real-time 3D multi-object detection and localization for both road and railway smart mobility. Based on a proven 2D real-time object detector, YOLOv3, our method offers encouraging results for real-time 3D detection. Our results also highlight the importance of accurate RoI prediction for all objects, especially for depth prediction. We tested our method on two datasets, the KITTI road dataset and our own hybrid virtual dataset (GTAV), including both road and railway images and scenes. The latter takes advantage of the graphics fidelity of the Grand Theft Auto V video game to offer a virtual dataset that is very close to reality. Finally, our results on our road/railway dataset show promising results. These results prove that our method can be used in the railway environment without loss of accuracy compared to road traffic scenes. We also plan to improve the accuracy and overall performance of our method through a refinement of the RoIs to improve their quality. The publication of the dataset as well as the code are planned in a future work. Finally, in addition to our virtual road/railway GTAV dataset, we are currently developing a new real road/railway dataset with ground truth, allowing us to go further in the development of not only the railway but also road e-ADAS. Our objective is to publish and share both virtual and real datasets in the fall of 2021. Our aim is to provide researchers and industry with an open platform including both datasets (virtual and real) so that they can experiment and validate their approaches for smart road and rail mobility. This will be the first such dataset in the world because today, no real road/rail dataset exists. There is only one dataset, which is dedicated to rail smart mobility, but it does not include ground truth such as RailSem19 [17]. We are going to replace YOLOv3 with YOLOv5 and will deeply modify our approach (our own network) to make it lighter (time and memory) and more accurate. We are going to use both virtual and real datasets to validate our new approach for 3D detection-based YOLOv5. We will reduce the accuracy gap of our method compared to the state-of-the-art approaches, and we will also carry out some experiments on an NVIDIA Jetson TX2embedded system dedicated to real-time artificial intelligence applications.

## Figures and Tables

**Table 1:** Quantitative results of our method for both KITTI and GTAV datasets. The evaluation was performed on the validation split of the datasets. Since accuracy metrics for the 2D detection (RoI) were not available for [11], only ours are displayed. The bold numbers represent the best score for each metrics for each dataset.

Dataset	Method	2D Detection				Distance							Dimensions DS	Center CS	Orientation OS	VRAM Usage	Inference Time
		AP	R	mAP	F1	Abs Rel	SRE	RMSE (m)	log RMSE	$\alpha_{1}$	$\alpha_{2}$	$\alpha_{3}$					
KITTI	Ours (w GT RoIs)	-	-	-	-	0.096	**0.307**	2.96	0.175	0.941	**0.980**	**0.988**	0.847	**0.983**	0.753	**3.22 GB**	**10 ms**
	Ours (w/o GT RoIs)	0.469	0.589	0.5	0.517	0.199	2.32	5.89	0.310	0.823	0.934	0.960	0.853	0.951	0.765	**3.22 GB**	**10 ms**
	Joint Monocular 3D [11]	-	-	-	-	**0.074**	0.449	**2.847**	**0.126**	**0.954**	**0.980**	0.987	**0.962**	0.918	**0.974**	5.64 GB	97 ms
GTA	Ours (w GT RoIs)	-	-	-	-	**0.069**	**0.420**	**4.60**	**0.100**	**0.965**	**0.992**	**0.999**	**0.886**	**0.999**	**0.961**	**3.22 GB**	**10 ms**
	Ours (w/o GT RoIs)	0.533	0.763	0.632	0.61	0.207	4.92	12.3	0.319	0.781	0.897	0.942	0.853	0.846	0.845	**3.22 GB**	

**Table 2:** Our experimental results for different object classes on both KITTI and GTAV datasets. The evaluation was performed on the validation split of the datasets. Since accuracy metrics for the 2D detection (RoI) were not available for [11], only ours are displayed. The bold numbers represent the best score for each metrics for each dataset.

Dataset	Classes	Method	2D Detection				Distance							Dimensions	Center	Orientation
			AP	R	mAP	F1	Abs Rel	SRE	RMSE (m)	log RMSE	$\alpha_{1}$	$\alpha_{2}$	$\alpha_{3}$	DS	CS	OS
KITTI	Car	Ours (w GT RoIs)	-	-	-	-	**0.0957**	0.312	3.05	0.18	**0.941**	0.980	0.988	**0.872**	0.981	0.781
		Ours (w/o GT RoIs)	0.558	0.879	0.783	0.682	0.163	1.29	5.28	0.271	0.839	0.948	0.971	0.867	0.962	**0.783**
	Person	Ours (w GT RoIs)	-	-	-	-	0.0979	**0.254**	**2.01**	0.154	0.935	**0.983**	**0.992**	0.705	0.993	0.584
		Ours (w/o GT RoIs)	0.508	0.518	0.464	0.513	0.424	7.92	7.70	0.485	0.729	0.844	0.892	0.719	0.885	0.616
	Cyclist	Ours (w GT RoIs)	-	-	-	-	0.0979	0.359	3.57	**0.150**	0.940	0.979	0.988	0.809	**0.997**	0.738
		Ours (w/o GT RoIs)	0.342	0.371	0.253	0.356	1.04	30.9	17.4	0.797	0.415	0.622	0.719	0.812	0.678	0.542
GTA	Car	Ours (w GT RoIs)	-	-	-	-	0.0552	0.385	4.74	0.0761	0.990	0.999	0.999	0.860	**0.999**	**0.993**
		Ours (w/o GT RoIs)	0.477	0.915	0.778	0.627	0.129	2.28	9.59	0.217	0.873	0.938	0.965	0.812	0.894	0.905
	Truck	Ours (w GT RoIs)	-	-	-	-	**0.0454**	**0.178**	**3.22**	**0.0576**	0.994	1.00	1.00	0.871	**0.999**	0.989
		Ours (w/o GT RoIs)	0.312	0.880	0.617	0.461	0.259	6.99	19.2	0.455	0.642	0.78	0.868	0.736	0.622	0.681
	Motorcycle	Ours (w GT RoIs)	-	-	-	-	0.0623	0.266	3.84	0.0753	**1.00**	**1.00**	**1.00**	0.918	1.00	0.967
		Ours (w/o GT RoIs)	0.614	0.764	0.725	0.681	0.186	3.58	13.7	0.281	0.691	0.878	0.967	0.901	0.689	0.909
	Person	Ours (w GT RoIs)	-	-	-	-	0.116	0.612	4.56	0.159	0.877	0.966	1.00	**0.963**	0.997	0.856
		Ours (w/o GT RoIs)	0.263	0.659	0.488	0.375	0.372	10.5	15.1	0.453	0.620	0.834	0.903	0.961	0.812	0.735

## Data Availability

The data that support the findings of this study are available from the corresponding author, A.M., upon reasonable request.

## Abbreviations

The following abbreviations are used in this manuscript:

ADAS	Advanced driver-assistance system
GTA	Grand Theft Auto
ToF	Time of flight
CNN	Convolutional neural networks
MLP	Multi-layer perceptron
PointRGCN	Pipeline based on graph convolutional networks
RoIs	Regions of interest
RPN	Region proposal network
NMS	Non-maximum suppression
GCN	Graph convolutional networks
ARE	Absolute relative error
RMSE	Root-mean-square error
BMP	Bad matching pixels
DS	Dimension score
CS	Center score
OS	Orientation score
YOLO	You only look once
LiDAR	Light detection and ranging
GT	Ground truth
